# Compact dual-band four-port MIMO patch antenna with inverse U-shaped resonators and defected ground structure for wireless communication applications

**DOI:** 10.1038/s41598-026-40390-0

**Published:** 2026-05-07

**Authors:** Ketavath Kumar Naik, Chirukuri Naga Phaneendra, Mohammad S. Zidan, Z. Zakaria, A. J. A. Al-Gburi

**Affiliations:** 1 Special Centre for Nanoscience, Jawaharlal Nehru University, New Delhi, 110067 India; 2 Department of ECE, Vignan’s Lara Institute of Technology and Science, Guntur, AP India; 3 Department of Electrical Techniques, Technical Institute of Anbar, Middle Technical University, Baghdad, 10074 Iraq; 4 Present Address: Center for Telecommunication Research & Innovation (CeTRI), Fakulti Teknologi Dan Kejuruteraan Elektronik Dan Komputer (FTKEK), Universiti Teknikal Malaysia Melaka (UTeM), Jalan Hang Tuah Jaya, Durian Tunggal, 76100 Malacca, Malaysia; 5 Strategic Research Institute (SRI), Asia Pacific University, Jalan Teknologi 5, Taman Teknologi Malaysia, 57000 Kuala Lumpur, Malaysia

**Keywords:** Compact size, Four-port MIMO antenna, Dual-band, MDGS, U-shaped slot, Wireless communication, Engineering, Physics

## Abstract

A compact four-port multiple-input multiple-output (MIMO) staircase rectangular patch antenna with inverse U-shaped resonators and a modified defected ground structure (MDGS) is presented for dual-band wireless communication applications. The antenna integrates four identical radiating elements placed orthogonally to achieve low mutual coupling and enhanced diversity. Fabricated on an FR-4 substrate with dimensions of 38 × 38 × 1.6 mm^3^, the design operates over two bandwidths (|S₁₁|< − 10 dB) of 6.8–8.9 GHz and 10.3–12.0 GHz, resonating at 7.9 GHz and 11 GHz, respectively. The structure achieves peak gains of 7.36 dBi and 5.82 dBi. MIMO performance is validated with an ECC below 0.01, DG greater than 9.95 dB, CCL below 0.02 bits/s/Hz, isolation exceeding 25 dB, mean effective gain (MEG) between − 9 dB and − 6 dB, and low TARC values. Simulated and measured results exhibit strong agreement, confirming the antenna’s potential for compact high-performance wireless devices within micro- and nanoscale integrated systems. The proposed design addresses key challenges in MIMO systems, such as minimizing mutual coupling, achieving wide dual-band operation, and maintaining compact size for integration in modern wireless devices.

## Introduction

Multiple-input multiple-output (MIMO) systems improve overall data throughput and boosts capacity in wireless communication systems. In this technology, number of antennas used to improve the overall system performance by simultaneously transmitting and receiving multiple data streams^1^. It utilizes the spatial domain to boost capacity, reliability, and overall wireless communication quality^2^. Advanced antenna systems outperform conventional monopoles by offering superior features and faster data speeds^3^, low attenuation, better channel capacity^4^, low latency^5^, low error rate and good diversity characteristics^6^. As a result, many researchers focus on designing MIMO antennas for wireless communication applications. Due to limited space available on substrate of MIMO system^6^, there is a chance of getting mutual coupling between elements. Several techniques aim to reduce mutual coupling between elements. Fractal crescent shape used for wireless applications^7^. The design of radiating element with insert-fed octagonal-structure proposed for wireless applications^8^. For X and Ku band wireless communications^9^, S-shaped slotted patch antenna fabricated. The tapered-fed MIMO model prototyped for the use of Ku band wireless applications with dual notch band characteristics^10^.

For X band wireless communication applications^11^, MIMO element with improved isolation^12^ is proposed. In^13,14^, concentric rectangular slot and dual polarized antenna reported for wireless radar applications. In^15,16^, different types of MIMO models are designed for wireless communication applications with better diversity performance. A MIMO antenna, featuring a flexible common patch design with four ports, serves wireless applications^17^. In^18^, a concise diversity setup featuring a U-shaped pattern offers superior gain^19^. MIMO antenna with a staircase quad-element design is proposed for wireless applications^20^. In^21^, a reconfigurable orthogonal four-port antenna^22^ for Ku band wireless applications. In^23^, CPW-fed antenna is designed for UWB. In^24^, an orthogonal microstrip feed line technique is utilized to ensure high isolation among MIMO elements intended for wireless applications^25^. A two-port MIMO antenna, structured in a rectangular ring shape^26^ modelled for wireless applications^27^. In^28^, a multiple-elements radiator with various circular slots is noted for generating high-gain characteristics^29^, ideal for wireless communication^30,31^. The substrate integrated waveguide MIMO antennas are also serving wideband wireless communication applications^32,33^. Different types of mode characteristics of common patch configuration are proposed for Wi-Fi 6 wireless applications^34,35^. The 4-port MIMO^36^ orthogonal-fed technique and massive MIMO antenna array^37,38^ are implemented to achieve pattern diversity applications.

The SRMP antenna with a staircase patch and U-shaped slots enables dual-band operation for wireless, radar, and satellite applications. With dimensions of 38 × 38 × 1.6 mm^3^, it achieves resonances at 7.9 GHz and 11.0 GHz, offering bandwidths of 2.1 GHz (6.8–8.9 GHz) and 1.7 GHz (10.3–12.0 GHz). Also, it has high gains of 7.36 dBi and 5.82 dBi, isolation > 25 dB, ECC < 0.01, and diversity gain > 9.95, providing robust multi-channel communication in space-constrained systems.

## Antenna design

**Fig. 1:**
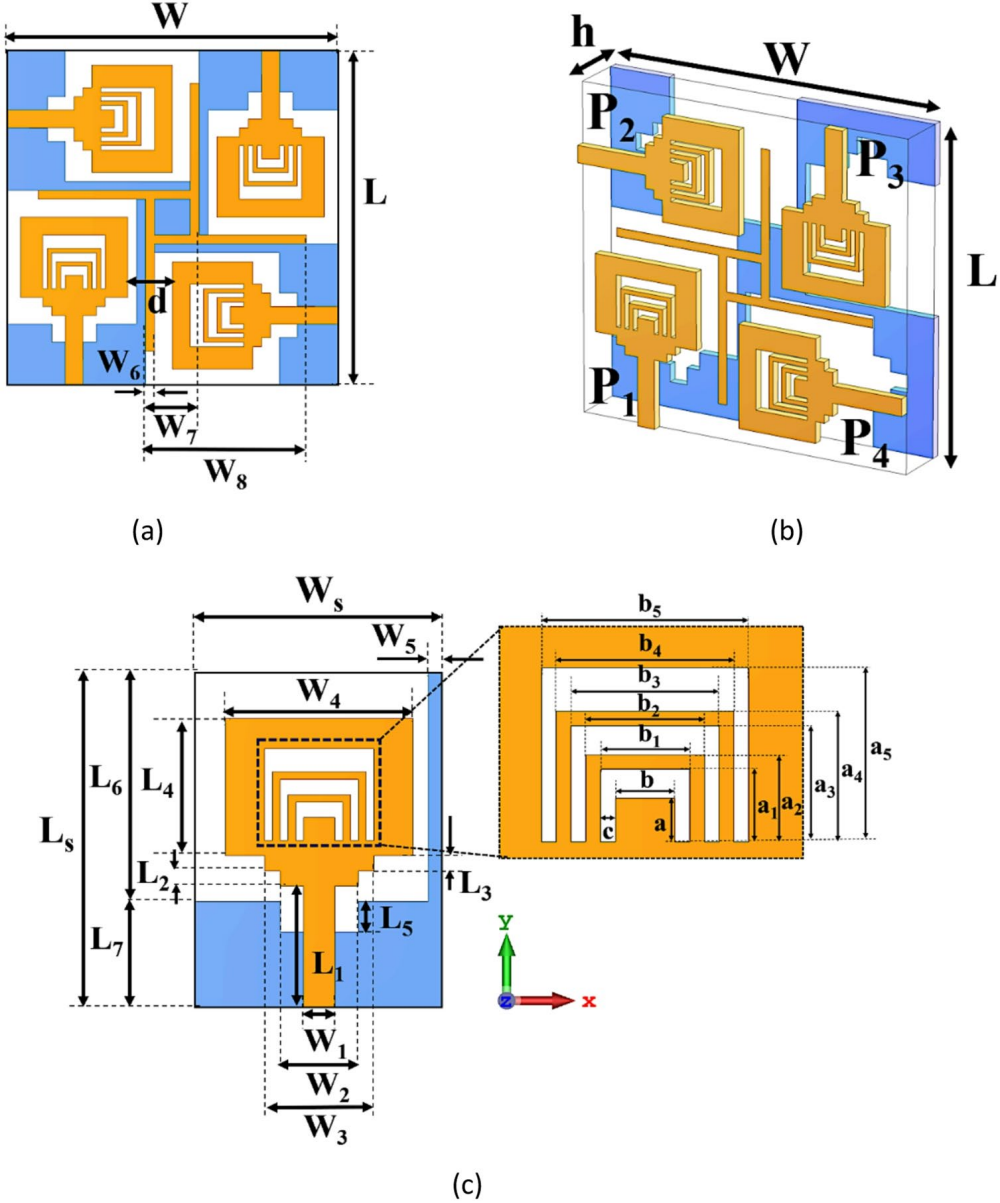
Dimensions of SRMP antenna (**a**) Top, (**b**) Perspective, (**c**) Unit element views.

A compact and novel staircase rectangular MIMO patch (SRMP) antenna with U shape slots and MDGS is designed for dual-band wireless communication. SRMPA is manufactured on low-cost FR-4 material with overall size of 38 × 38 × 1.6 mm^3^. Fr-4 material having dielectric constant (Ɛ_*r*_ = 4.4) and lossless tangent (*δ* = 0.02). In order to obtained the 50-Ω impedance matching rectangular shape staircase structure is proposed. The physical dimensions of each parameter used in design process of SRMP antenna with different views are illustrated in Fig. 1a–c. Optimum design specifications of SRMPA are listed in Table 1.

**Table 1:** Design specifications of the SRMP antenna.

Variables	Magnitude (mm)	Variables	Magnitude (mm)	Variables	Magnitude (mm)
L	38	W_1_	2	a_3_	4
W	38	W_2_	5	a_4_	4.5
L_S_	22	W_3_	7	a_5_	6
W_S_	16	W_4_	12	b	2
L_1_	8	W_5_	1	b_1_	3
L_2_	1	W_6_	1	b_2_	4
L_3_	1	W_7_	6	b_3_	5
L_4_	9	W_8_	18	b_4_	6
L_5_	2	a	1.5	b_5_	7
L_6_	15	a_1_	2.5	c	0.5
L_7_	7	a_2_	3	h	1.6

The SRMP antenna has ports designated as P1, P2, P3, and P4. Dimensions of basic rectangular patch radiating element is obtained by using Eqs. (1, 2) ^9^.

1 $${\mathrm{W}} = { }\frac{{\mathrm{C}}}{{2{\mathrm{f}}_{{\mathrm{r}}} }}\sqrt {\frac{2}{{{\upvarepsilon }_{{\mathrm{r}}} + 1}}}$$

2 $${\mathrm{L}} = { }\left\{ {\frac{{\mathrm{C}}}{{2{\mathrm{f}}_{{\mathrm{r}}} \sqrt {{\upvarepsilon }_{{\text{r eff}}} } }}} \right\}$$

Effective dielectric constant, ε_reff_, is estimated using:

3 $${\upvarepsilon }_{{\mathrm{reff}}} = { }\frac{{{\upvarepsilon }_{{\mathrm{r}}} + 1}}{2} + { }\frac{{{\upvarepsilon }_{{\mathrm{r}}} - 1}}{2}\left[ {\left( {1 + { }\frac{{12{\mathrm{t}}}}{{\mathrm{W}}}} \right)^{{ - \frac{1}{2}}} + 0.04{ }\left( {1 - { }\frac{{\mathrm{W}}}{{\mathrm{t}}}} \right)^{2} } \right]$$

where Ɛ_*r*_ is relative dielectric constant, and *f*_*r*_ is resonant frequency.

## Design process and analysis of SRMP antenna

### Antenna development

**Fig. 2:**
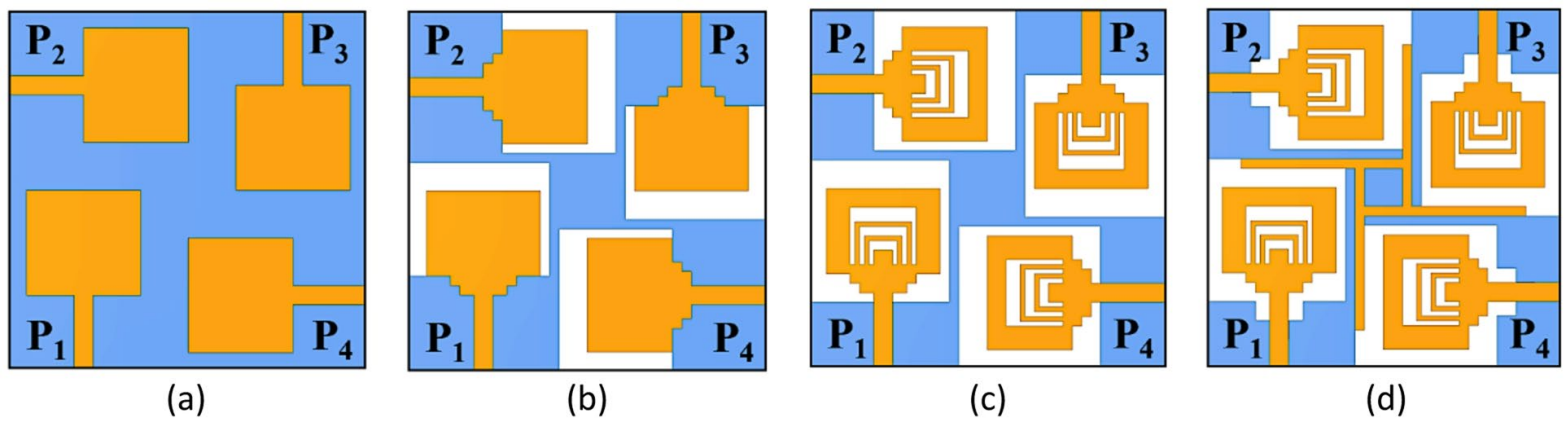
Design process of SRMP antenna (**a**) Ant.1, (**b**) Ant.2, (**c**) Ant.3, (**d**) Ant.4.

The design process of SRMP antenna is presented by considering MIMO structure step-by-step. Step 1, The SRMP antenna starts with a rectangular patch and ground plane, labelled Ant.1 in Fig. 2a. Dimensions of rectangular patch are derived from Eq. (1, 2) and resonating at 12 GHz with a minimum S_11_ of − 13 dB. Ant.1 has 3.56dBi low gain and 400 MHz bandwidth. In the next step, to resonate SRMP antenna within the band, produce dual-band characteristics and for impedance matching rectangular-patch converted into staircase rectangular-patch with modified defected ground structure (MDGS) is called as Ant.2 presented in Fig. 2b. Ant.2, resonating within band 10 GHz and 12.3 GHz with minimum reflection coefficients of − 16 dB and − 15 dB respectively. Ant.2 has gain of 4.98dBi and 4.51dBi at resonating frequencies.

After that, enhance the S_11_, and bandwidth U shape slots are etched from radiating patches with a reduced height of MDGS called as Ant.3 shown in Fig. 2c. Ant.3, resonating within the band at 7.2 GHz and 10.6 GHz with reflection coefficients of − 31 dB and − 21 dB respectively. Ant.3 has a gain of 3.96dBi and 4.48dBi at resonating frequencies. However, Ant.3 has low gain compared to Ant.2, low isolation characteristics, high mutual coupling low diversity performance. To reduce mutual coupling and improve diversity, ground and decoupling structure (1 × 17 mm^2^) are modified, resulting in Ant.4 shown in Fig. 2d.

**Fig. 3:**
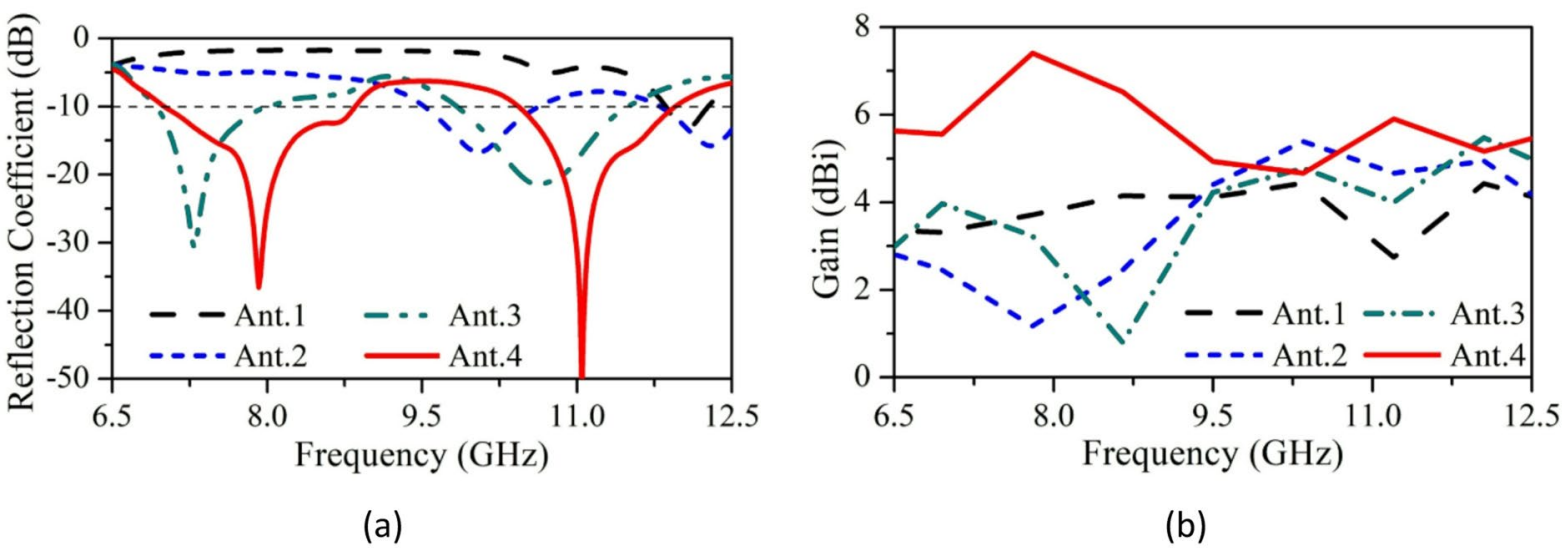
Evolution process of SRMP antenna with respect to (**a**) S_11_, (**b**) Gain.

Ant.4 (SRMP antenna), operating at 7.9 GHz and 11 GHz with high gain, high bandwidth, and S_11_ of 7.36dBi, 5.82dBi, 2.1 GHz, 1.7 GHz, − 37 dB and − 52 dB respectively. Evolution of SRMP antenna with respect to S_11_ and gain characteristics are shown in Fig. 3a, b. Quantitative analysis of SRMPA is listed in Table 2.

**Table 2:** Quantitative analysis of SRMP antenna.

Evolution	Resonating frequency (GHz)	Gain (dBi)	Bandwidth (MHz)	S_11_ (dB)
Ant. 1	12	3.56	400	− 13
Ant. 2	10	4.98	1000	− 16
	12.3	4.51	800	− 15
Ant. 3	7.2	3.96	1300	− 31
	10.6	4.48	1650	− 21
Ant. 4	7.9	7.36	2100	− 37
	11	5.82	1700	− 52

**Fig. 4:**
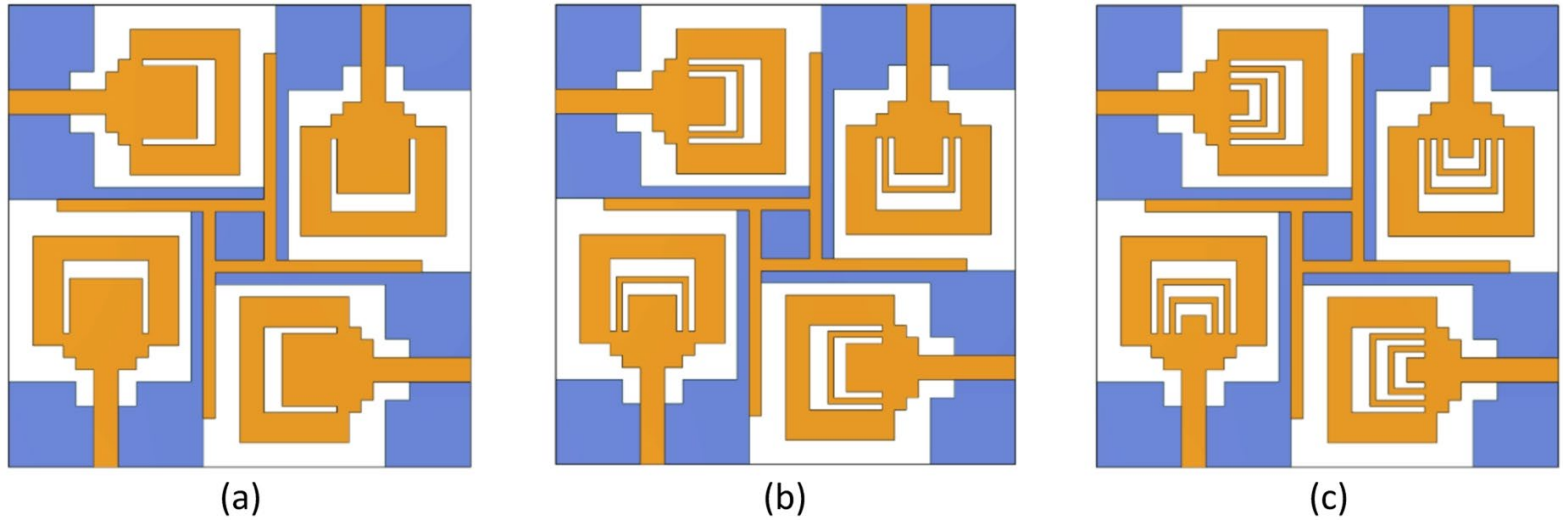
SRMP antenna with inverse U-shaped slots of (**a**) Single, (**b**) Double, and (**c**) Triple.

**Fig. 5:**
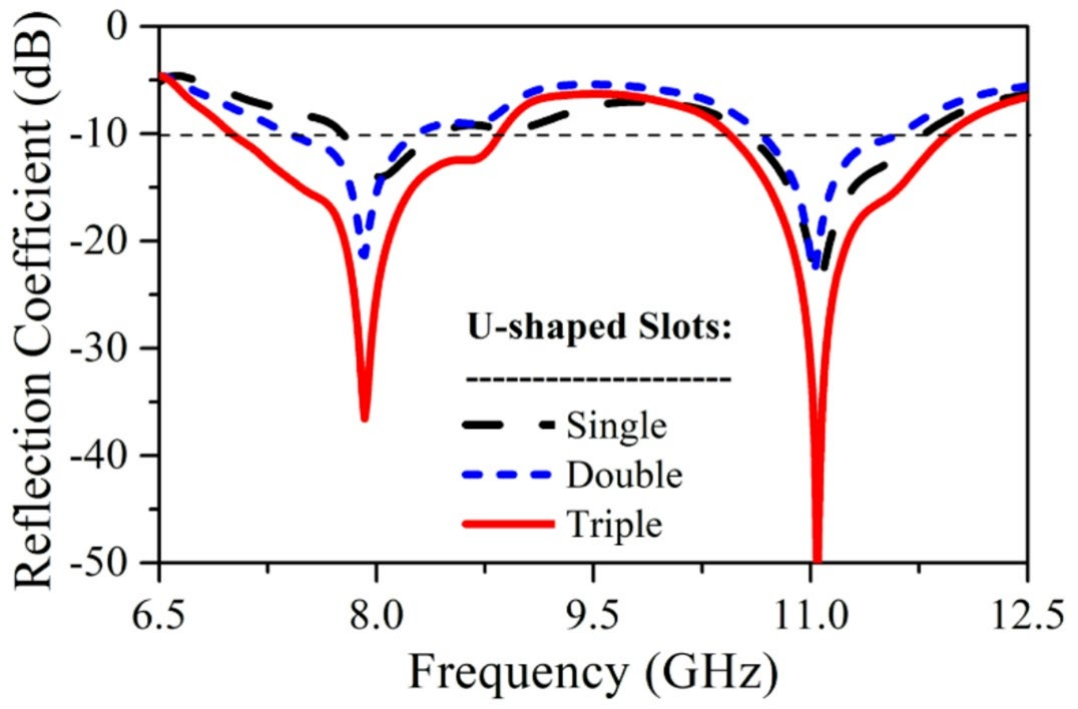
SRMP antenna S_11_ characteristics with respect to no. of slots.

Here, the analysis is carried out for the proposed SRMP antenna with respect to inverse U-shaped slots was proposed as single, double, and triple slots and also presented in Fig. 4a–c respectively. Figure 5 shows the corresponding reflection coefficient (S_11_) characteristics of the proposed SRMP antenna as single, double, and triple U-shaped slots. The comparison of proposed as single, double, and triple slots with respect to resonating frequency, gain and S11 is presented in the below Table 3.

**Table 3:** The comparison of proposed as single, double, and triple slots.

No. of U-slots	Resonating frequency (GHz)	Gain (dBi)	S_11_ (dB)
Single	8.1	3.22	− 15
	10.9	4.54	− 25
Double	7.85	4.38	− 24
	11	4.62	− 25
Triple	7.9	7.36	− 37
	11	5.82	− 52

In the proposed SRMP antenna, each inverse U-shaped slot contributes differently to the overall current distribution and resonance behavior due to its width variation. When we insert First U-shaped slot (single) shown in Fig. 4a, the SRMP antenna resonates at higher frequency (~ 11 GHz) and improving impedance matching and the gain and S_11_ is presented in the below table. The Second U-shaped slot (double) inserted shown in Fig. 4b, then the SRMP antenna provides stronger coupling to the patch currents and is mainly responsible for generating (dual resonance) another resonance (~ 7.9 GHz) along with first resonance (~ 11 GHz) by creating a significantly longer current path. When we insert the third U-shaped slot shown in Fig. 4c, the SRMP antenna redistributes the surface currents, stabilizes the dual-band response, provides wider impedance bandwidth, and enhances isolation by suppressing parallel coupling currents across the patch. The corresponding reflection coefficient (S_11_) characteristics are shown in Fig. 5 and Table 3. From that analysis it is clear that all three inverse U-shaped slots having individual significant contribution in the design of proposed SRMP antenna. So, we proposed SRMP antenna is in the final design with triple inverse U-shaped slots.

### Step-by-step optimization process

**Fig. 6:**
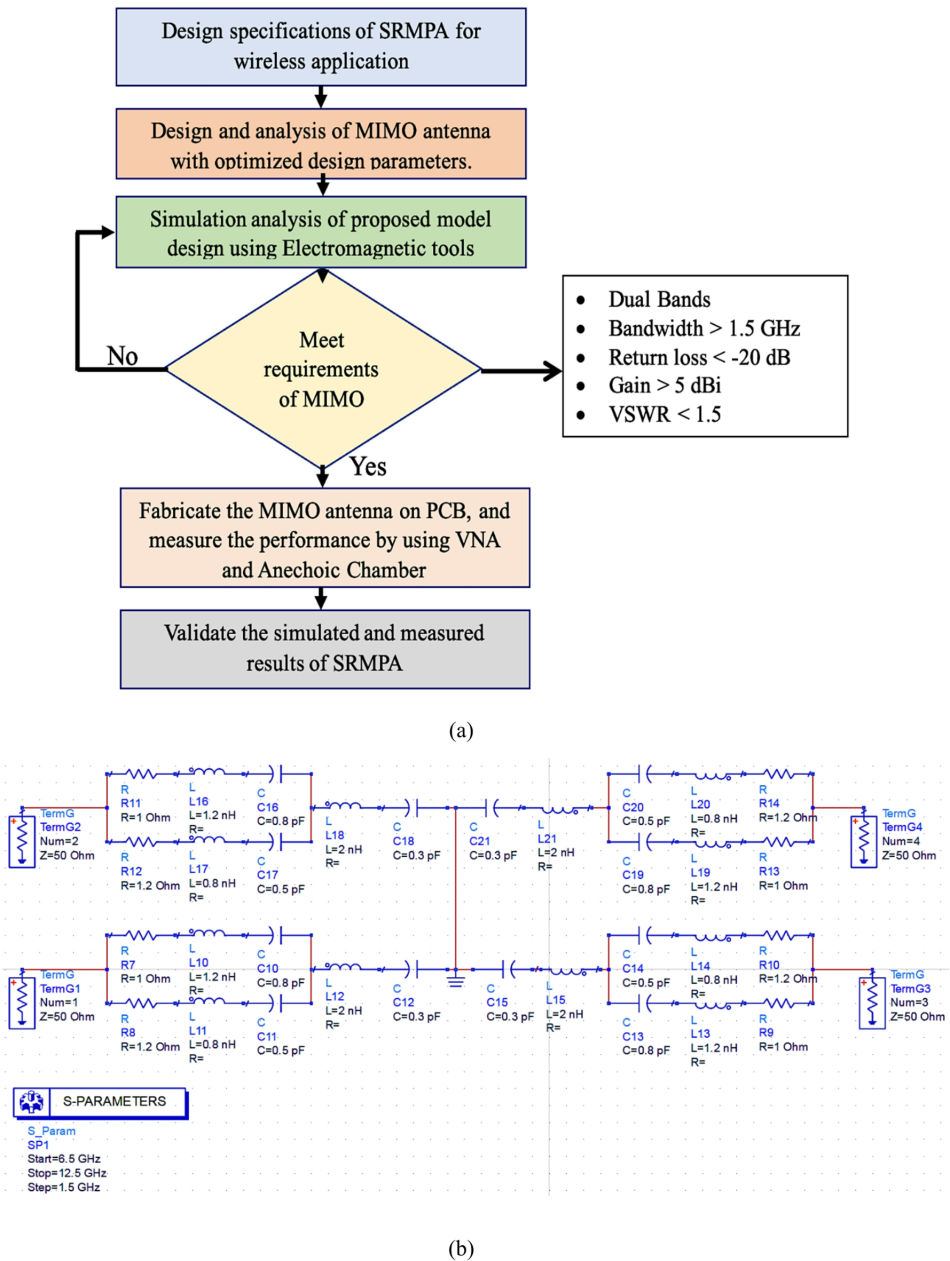
SRMP antenna (**a**) Design methodology flow diagram, (**b**) Equivalent circuit model.

The proposed SRMP antenna is designed based on methodology given above shown in Fig. 6a. Initially starts with identifying the problem and defining the requirements for the proposed wireless applications, including operating frequency, bandwidth, gain, and isolation. The substrate material is selected based on its dielectric constant, loss tangent, and thickness, which significantly impact antenna performance. Patch dimensions are calculated using transmission line formulas to ensure resonance at the desired frequency. Simulation tools such as HFSS or CST are then employed to design and analyze SRMP antenna parameters, including S-parameters, radiation patterns, and gain, with iterative adjustments for optimization. Isolation between ports is improved by modified defected ground structure (MDGS). Once the SRMP antenna meets the proposed application’s requirements after optimization, it is fabricated using PCB etching, and connectors are soldered at the feed points. The SRMP antenna results and MIMO parameters are validated using VNA and anechoic chamber. The equivalent circuit model of SRMP antenna is presented in Fig. 6b. Each antenna port is modeled using two parallel RLC resonators, which replicate the resonances at 7.9 GHz and 11.0 GHz introduced by the staircase patch and U-shaped slots. These RLC branches emulate the antenna’s impedance characteristics, where inductors represent current paths, capacitors account for slot effects, and resistors model radiation and material losses. A parallel LC tank circuit models the modified defected ground structure (MDGS), which serves as a band stop filter to suppress surface currents and reduce mutual coupling, thereby enhancing isolation and impedance matching. The ports are terminated with standard 50 Ω loads, and all branches are grounded through the MDGS network, completing the return path and accurately reflecting the antenna’s real-world operation. The SRMP antenna resonates in two bands and the resonance frequency is obtained using below derived Eqs. (4–5). The resonance frequency was presented with RLC circuit analysis of the proposed SRMP antenna.

4 $$f_{r1} = \frac{C}{{2 L\sqrt {\varepsilon_{eff} } }} \left( {\frac{1 - A}{{1 + A \left( {\ln \left( {\frac{1.123L}{{\sqrt {\varepsilon_{eff} } h}}} \right)} \right)}}} \right)\;{\mathrm{and}}\;f_{r2} = \frac{C}{{2L \sqrt {\varepsilon_{eff} } }}$$

here, the A is constant, L is inductance and C is capacitance

5 $$A = \frac{2}{{\pi \varepsilon_{eff} }} \left( {\frac{L}{h} + 1.393 + 0.667\ln \left( {\frac{L}{h} + 1.444} \right)} \right)$$

### Parametric analysis

In order obtain, the optimum values of SRMP antenna design parameters through various iterations parametric analysis are used. Parametric analysis of SRMPA is done using CST. This paper analyses the impact of parameters on frequency, gain, and bandwidth to optimize design.

**Fig. 7:**
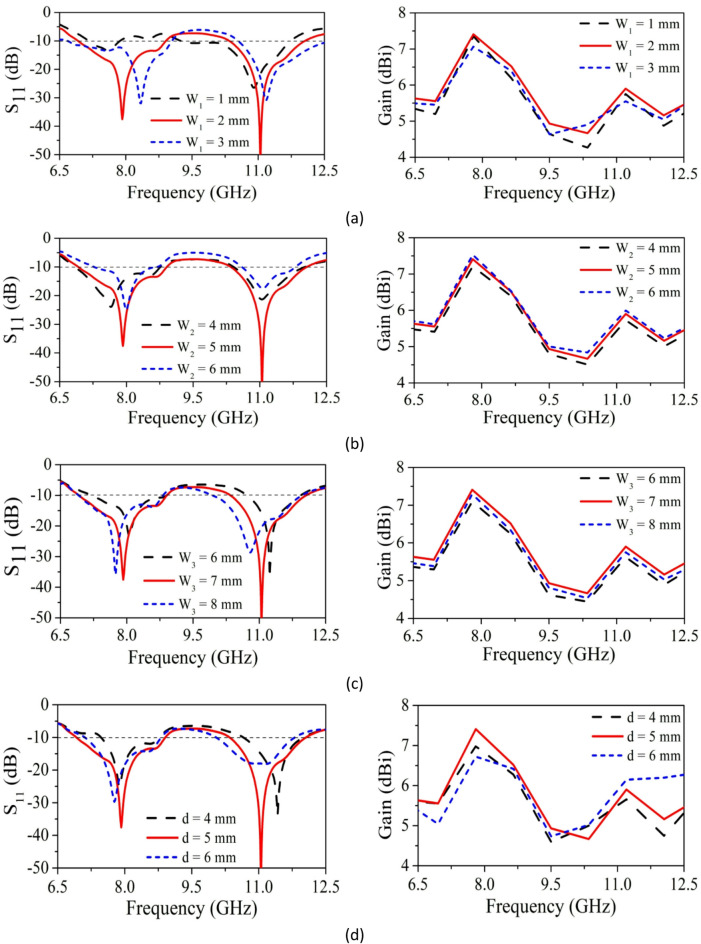
Parametric analysis of SRMA width parameters (**a**) W_1_, (**b**) W_2_, (**c**) W_3_, (**d**) d.

*Influence of the Parameter (W*_*1*_*)* W_1_ (feed line width) is varied from 1 to 3 mm in 1 mm steps to analyze its impact on SRMPA performance. When W_1_ = 1 mm, the SRMPA resonating single band at 11 GHz with S_11_ of − 26 dB. Also, it is having 1.66 GHz bandwidth and 5.74dBi gain. When W_1_ = 2 mm, the SRMP antenna provides dual-band characteristics at 7.9 GHz and 11 GHz with S_11_ of − 37 dB and − 52 dB. Also, it is having gain and bandwidth of 7.36dBi, 5.82dBi, 2.6 GHz, 1.7 GHz respectively. When W_1_ = 3 mm, the SRMP antenna exhibits dual-band at 8.2 GHz and 11.1 GHz, with S_11_ of − 32 dB and − 30 dB. Also, it is having gain and bandwidth of 6.8dBi, 5.71dBi, 2.6 GHz, 1.7 GHz. The optimum values of SRMP antenna attained when we consider W_1_ = 2 mm. Hence, optimum value of parameter W_1_ is 2 mm shown in Fig. 7a.

*Influence of the Parameter (W*_*2*_*)* To know the impact of parameter W_2_ (width of rectangular slit in MDGS) on performance of SRMP antenna, W_2_ ranges from 4 to 6 mm with step size of 1 mm. When W_2_ = 4 mm and 6 mm, the SRMP antenna provide dual band characteristics. From the analysis shown in Fig. 7b, S_11_ for 4 mm and 6 mm is low compared to 5 mm, bandwidth is also low, gain deviations are minute. Optimum value of W_2_ is 5 mm, based on S_11_ and bandwidth.

*Effect of parameter (W*_*3*_*)* W_3_ (staircase step 2) is varied from 6 to 8 mm. At W_3_ = 6 mm and 8 mm, dual-band is observed, but with lower bandwidth and higher gain deviation compared to 7 mm. The optimum W_3_ value is 7 mm based on S_11_, bandwidth, and gain is illustrated in Fig. 7c.

*Effect of parameter (d)* d (spacing between MIMO elements) parameter impact on performance of SRMPA is validated by varying from 4 to 6 mm with step size of 1 mm. When d = 4 mm and 6 mm, the SRMP antenna provide dual band characteristics with frequency shifting. From analysis is shown in Fig. 7d, S_11_ for 4 mm and 6 mm is low compared to 5 mm, bandwidth is also low, gain deviations are also minute. Based on better reflection coefficient, bandwidth, and gain optimum value of parameter d is considered as 5 mm.

**Fig. 8:**
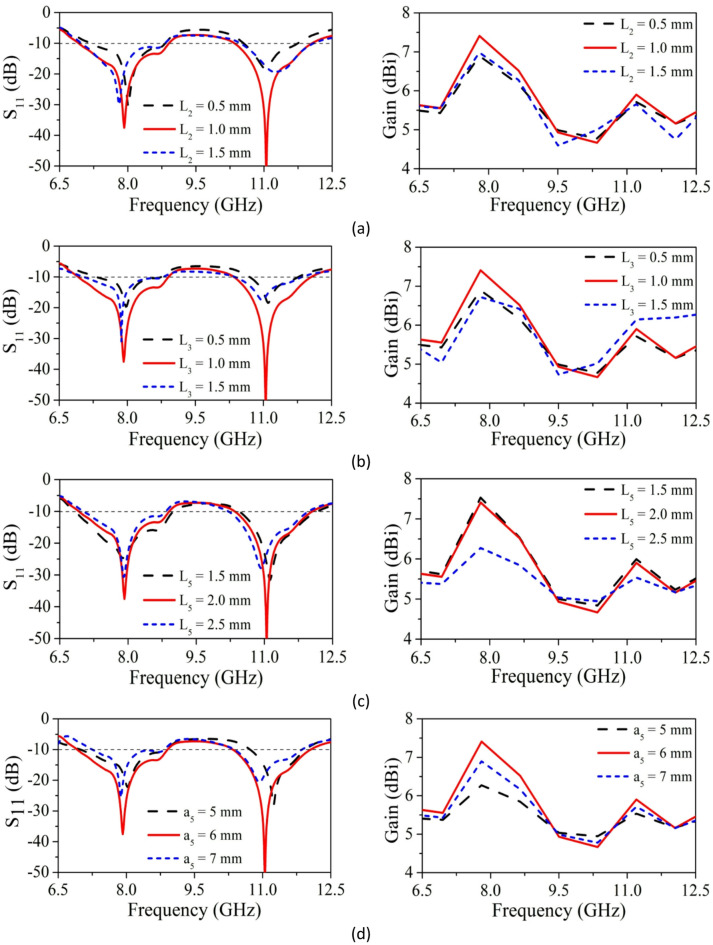
Parameter-based analysis of SRMPA length parameters (**a**) L_2_, (**b**) L_3_, (**c**) L_5_, (**d**) a_5_.

*Effect of Parameter L*_*2*_ L_2_ (staircase step-1 length) parameter impact on performance of SRMPA is validated by varying from 0.5 mm to 1.5 mm with step size of 0.5 mm. When L_2_ = 0.5 mm and 1.5 mm, SRMP antenna provides dual-band characteristics at 8.0 GHz, 11.0 GHz, 7.8 GHz, and 11.3 GHz with reflection coefficients of − 30 dB, − 15 dB, − 29 dB, and − 16 dB. It is also having gain of 6.0dBi, 5.5dBi, 6.6dBi, and 5.4dBi. When L_2_ = 1.0 mm, SRMP antenna resonating at 7.9 GHz and 11.0 GHz with reflection coefficient of − 37 dB and − 52 dB. Also, it is having high gain of 7.36dBi and 5.82dBi. From the analysis depicted in Fig. 8a, The optimum characteristics of SRMP antenna is attained when L_2_ = 1.0 mm. Hence, the optimum value of parameter L_2_ is considered as 1.0 mm.

*Effect of Parameter L*_*3*_ To further examine the influence the of parameter L_2_ (staircase step-2 length) on performance of SRMP antenna, parameter “L_3_” varied from 0.5 mm to 1.5 mm with step size of 0.5 mm. When we consider L_3_ = 0.5 mm and 1.5 mm, the SRMP antenna provides dual-band characteristics at 8.0 GHz, 11.1 GHz, 7.9 GHz, and 11.0 GHz with reflection coefficients of − 19 dB, − 17 dB, − 32 dB, and − 15 dB. It is also having gain of 6.2dBi, 5.6dBi, 6.4dBi, and 5.6dBi. The bandwidth, return loss, and gain values of above two cases are low when we compared with L_3_ = 1.0 mm. From Fig. 8b, optimum value for L_3_ is 1.0 mm.

*Effect of Parameter L*_*5*_ To examine the influence of parameter L_5_ (depth of ground slit) on performance of SRMP antenna, parameter “L_5_” varied from 1.5 mm to 2.5 mm with step size of 0.5 mm. When we consider L_5_ = 1.5 mm, L_5_ = 2.0 mm, and L_5_ = 2.5 mm, we observed minute resonating frequency shift, band width remains same for all three cases. However, the gain of L_5_ = 2.0 mm is high when compared to the remaining two cases. From the analysis shown in Fig. 8c, The optimum results of SRMP antenna are attained L_5_ = 2.0 mm. Hence, the optimum value of parameter L_5_ is considered as 2.0 mm.

*Effect of parameter (a*_*5*_*)* a (height of inverse U-shaped slot on rectangular patch) parameter impact on the performance of SRMPA is calculated by varying it is from 5 to 7 mm with a step size of 1 mm. When we consider a_5_ = 5 mm and 7 mm, the SRMP antenna provides dual-band characteristics at 8.1 GHz, 11.2 GHz, 7.9 GHz, and 11 GHz with reflection coefficients of − 20 dB, − 28 dB, − 25 dB, and − 19 dB. It is also having gain of 6.2dBi, 5.4dBi, 6.9dBi, and 5.7dBi. These values are low when we compared with a_5_ = 6 mm. From the analysis shown in Fig. 8d, The optimum characteristics of SRMPA is attained when a_5_ = 6 mm. Therefore, the optimum value of parameter a_5_ is found to be 6 mm.

### MIMO performance metrics

**Fig. 9:**
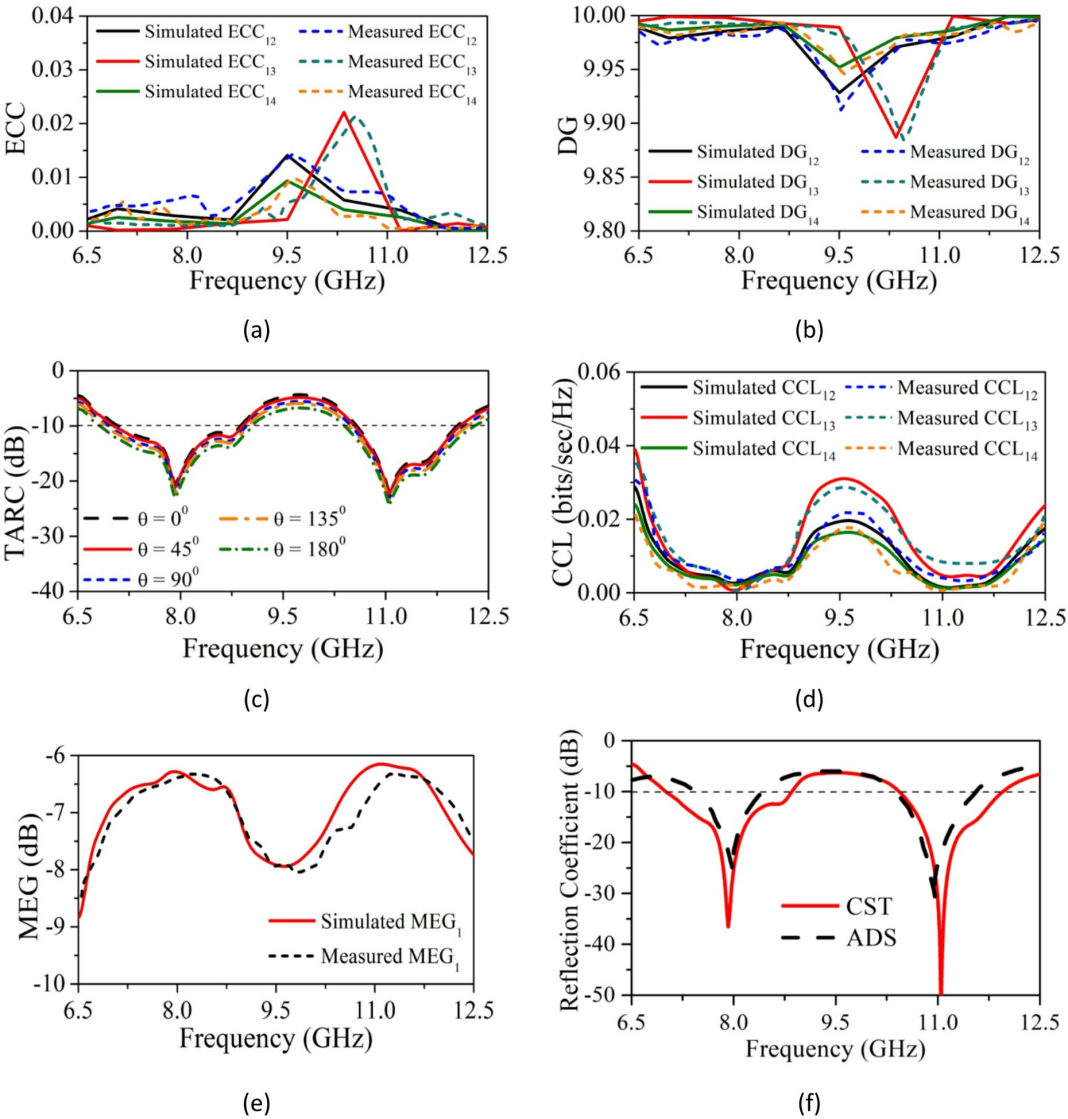
Predicted and tested (**a**) ECC, (**b**) DG, (**c**) TARC, (**d**) CCL, (**e**) MEG and (**f**) S_11_ comparison of both CST & ADS software.

To assess SRMP antenna diversity, ECC and DG are analyzed and shown in Fig. 9a, b. ECC is calculated from far-field patterns, and DG from Eqs. (6) and (7) ^7,35^. The ideal values are ECC < 0.5 and DG > 9.5 ^7^. The SRMP antenna obtains ECC < 0.01 and DG > 9.95 in the resonating band.

6 $$\rho_{e} = \frac{{\left| {\mathop \smallint \nolimits_{0}^{2\pi } \mathop \smallint \nolimits_{0}^{\pi } \left( {XPRE_{\theta 1} E_{\theta 2}^{*} P_{\theta } + E_{\varphi 1} E_{\varphi 2}^{*} P_{\varphi } } \right) d\Omega } \right|^{2} }}{{\mathop \smallint \nolimits_{0}^{2\pi } \mathop \smallint \nolimits_{0}^{\pi } \left( {XPRE_{\theta 1} E_{\theta 1}^{*} P_{\theta } + E_{\varphi 1} E_{\varphi 1}^{*} P_{\varphi } } \right) d\Omega X \mathop \smallint \nolimits_{0}^{2\pi } \mathop \smallint \nolimits_{0}^{\pi } \left( {XPRE_{\theta 2} E_{\theta 2}^{*} P_{\theta } + E_{\varphi 2} E_{\varphi 2}^{*} P_{\varphi } } \right) d\Omega }}$$

7 $$DG = 10\sqrt {1 - \left[ {\rho_{e} } \right]^{2} }$$

where, θ and φ represent vertical and horizontal polarized patterns of SRMPA, XPR is cross-polar discrimination, and ECC i,j and DG i,j indicate correlation and diversity gain between ports i and j. Due to symmetry, ECC i,j = ECC j,i and DG i,j = DG j,i.

The SRMPA active bandwidth is calculated employing TARC Eq. (8) (Fig. 9c). CCL, calculated from Eq. (9) and shown in Fig. 9d ^7^, is ideally < 0.5 bits/sec/Hz. The SRMPA achieves CCL < 0.02 bits/sec/Hz, indicating high diversity performance.

8 $$\Gamma_{{\mathrm{a}}}^{{\mathrm{t}}} = \frac{{\sqrt {\begin{array}{*{20}c} {\left| {\left( {{\mathrm{S}}_{11} + {\mathrm{S}}_{12} {\mathrm{e}}^{{{\mathrm{j}}{ theta }}} + {\mathrm{S}}_{13} {\mathrm{e}}^{{{\mathrm{j}}{ theta }}} + {\mathrm{S}}_{14} {\mathrm{e}}^{{{\mathrm{j}}{ theta }}} } \right)} \right|^{2} + \left| {\left( {{\mathrm{S}}_{21} + {\mathrm{S}}_{22} {\mathrm{e}}^{{{\mathrm{j}}{ theta }}} + {\mathrm{S}}_{23} {\mathrm{e}}^{{{\mathrm{j}}{ theta }}} + {\mathrm{S}}_{24} {\mathrm{e}}^{{{\mathrm{j}}{ theta }}} } \right)} \right|^{2} } \\ { + \left| {\left( {{\mathrm{S}}_{31} + {\mathrm{S}}_{32} e^{{{\mathrm{j}}{ theta }}} + {\mathrm{S}}_{33} {\mathrm{e}}^{{{\mathrm{j}}{ theta }}} + {\mathrm{S}}_{34} {\mathrm{e}}^{{{\mathrm{j}}{ theta }}} } \right)} \right|^{2} + \left| {\left( {{\mathrm{S}}_{41} + {\mathrm{S}}_{42} {\mathrm{e}}^{{{\mathrm{j}}{ theta }}} + {\mathrm{S}}_{43} {\mathrm{e}}^{{{\mathrm{j}}{ theta }}} + {\mathrm{S}}_{44} {\mathrm{e}}^{{{\mathrm{j}}{ theta }}} } \right)} \right|^{2} } \\ \end{array} } }}{2}$$

9 $$CCL = - log_{2 } \det \left[ {\begin{array}{*{20}c} {\begin{array}{*{20}c} {\beta_{11} } & {\beta_{12} } \\ {\beta_{21} } & {\beta_{22} } \\ \end{array} } & {\begin{array}{*{20}c} {\beta_{13} } & {\beta_{14} } \\ {\beta_{23} } & {\beta_{24} } \\ \end{array} } \\ {\begin{array}{*{20}c} {\beta_{31} } & {\beta_{32} } \\ {\beta_{41} } & {\beta_{42} } \\ \end{array} } & {\begin{array}{*{20}c} {\beta_{33} } & {\beta_{34} } \\ {\beta_{43} } & {\beta_{44} } \\ \end{array} } \\ \end{array} } \right]$$

where, $${\beta }_{ii}= 1- \left({\left|{S}_{ii}\right|}^{2}+ {\left|{S}_{ij}\right|}^{2}\right)$$ & $${\beta }_{ij}= - \left({S}_{ii}^{*}{S}_{ij}+{S}_{ji}^{*}{S}_{ij}\right)$$

The comparison of S-parameters with respect to CST and ADS software is shown in Fig. 9f. The MEG of the SRMPA with ‘n’ ports is derived from S-parameters (Eq. 10)^7,36^. Simulated and measured MEG_1_ ranges from − 6 dB to − 9 dB, as shown in Fig. 9e.

10 $$MEG_{i} = 0.5 \left[ {1 - \mathop \sum \limits_{j = 1}^{n} \left| {S_{ij} } \right|^{2} } \right]$$

where, i and j are port numbers.

Simulated and measured values of port 1 (P_1_) is only presented in Fig. 9e. MEG at P_2_, P_3_, and P_4_ are almost same as P_1_ because of identical structure.

## Experimental results and discussion

**Fig. 10:**
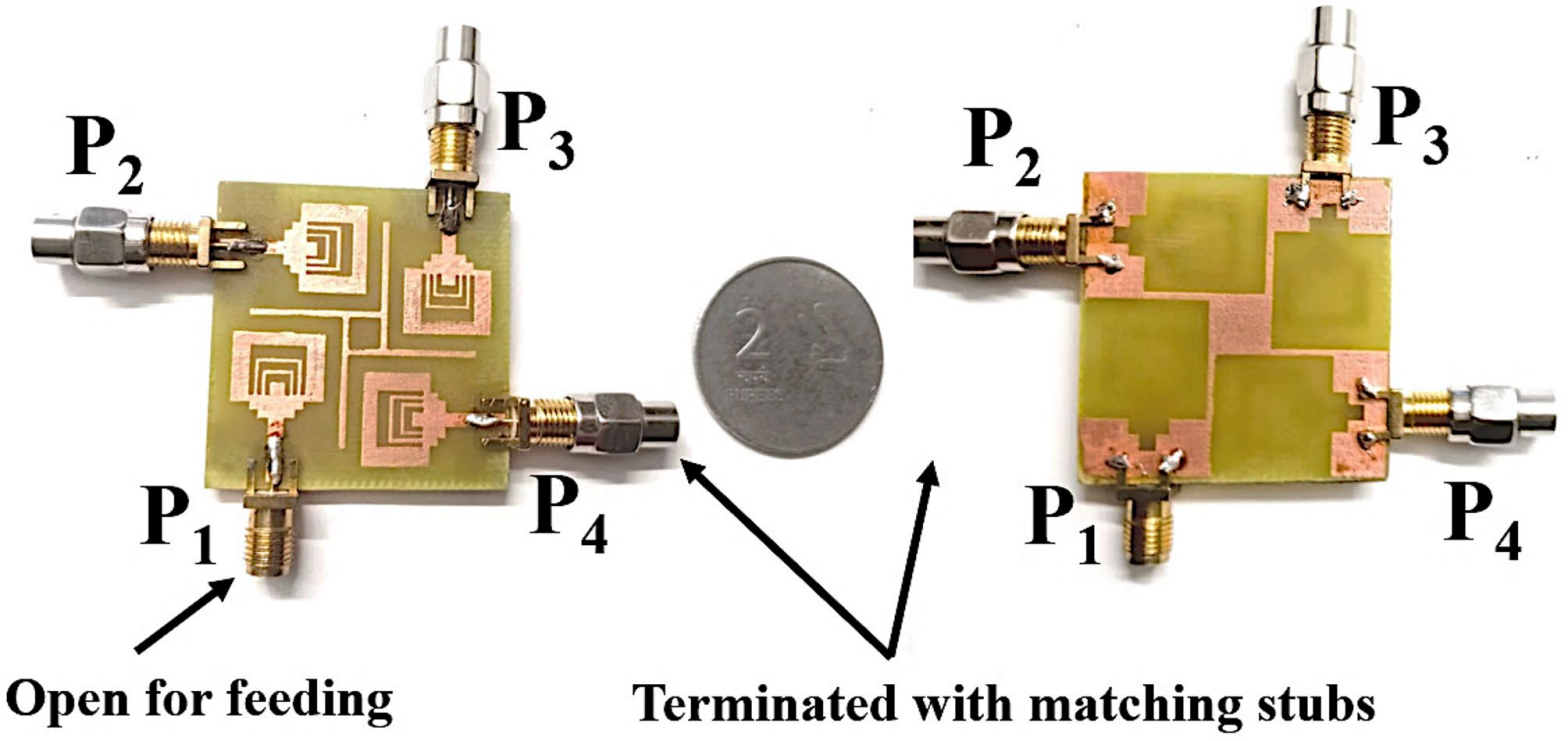
Front and ground views of SRMP antenna physical prototype.

**Fig. 11:**
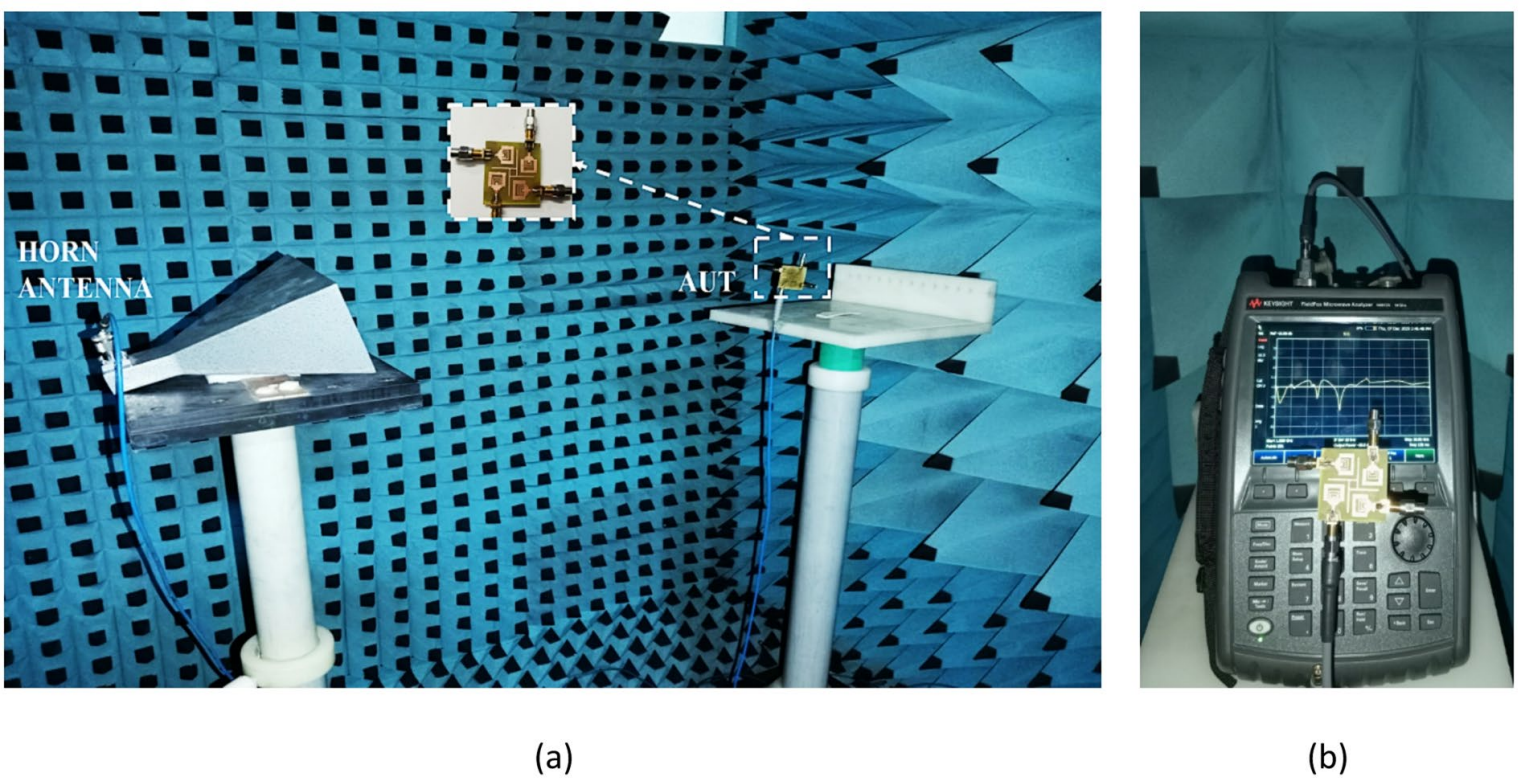
Measurement setup of SRMPA (**a**) AUT measurement setup (**b**) VNA results.

**Fig. 12:**
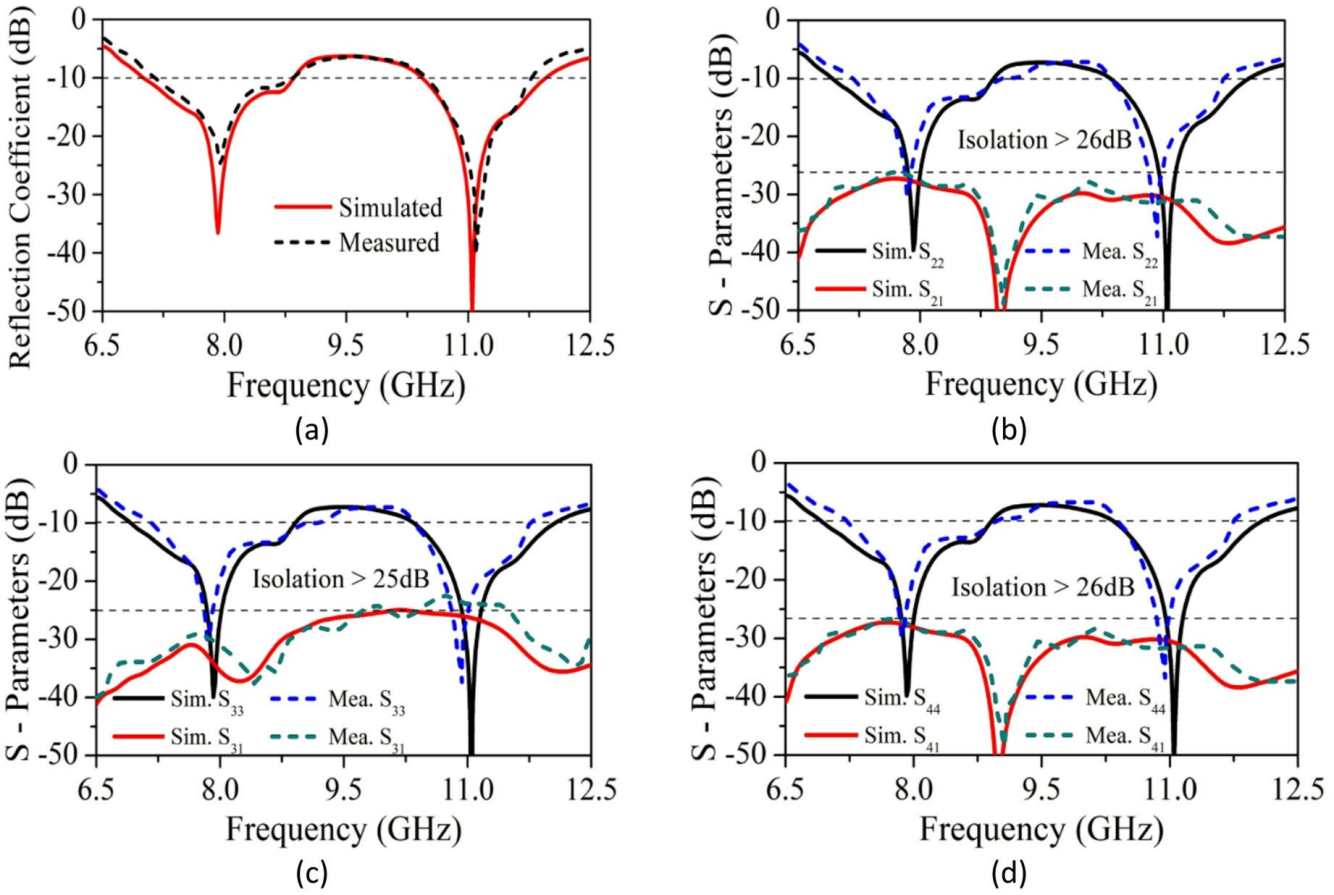
Results of SRMPA (**a**) S_11,_ (**b**) S_22_ & S_21,_ (**c**) S_33_ & S_31,_ (**d**) S_44_ & S_41,_

The SRMPA is modelled and analyzed using CST software. To compare practical results with simulations, a prototype is fabricated (Fig. 10). Measured characteristics are obtained using a VNA (N9917A), with port-1 excited and other ports terminated with a 50-Ω load. Measurement setup in an anechoic chamber and S_11_ characteristics on VNA are illustrated in Fig. 11a, b for validation. The simulated and experimental S_11_ of SRMPA is shown in Fig. 12a. Scattering parameters S_22_ and S_21_ are presented in Fig. 12b, S_33_ and S_31_ in Fig. 12c, and S_44_ and S_41_ in Fig. 12d.

**Fig. 13:**
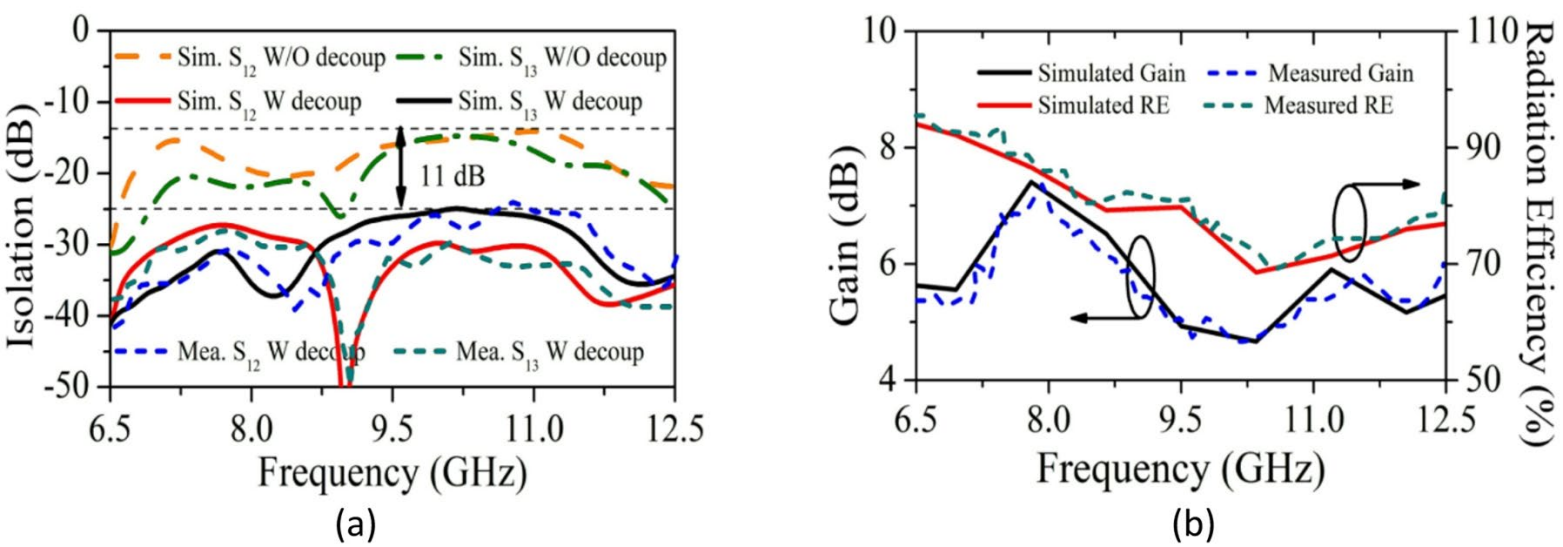
Predicted and experimental (**a**) S_12_, S_13_, S_14,_ (**b**) Gain and RE.

Figure 13a indicates simulated and practical isolation of P_1_-P_2_ and P_1_-P_3_, with and without the decoupling structure. Adding the configuration increases isolation to 11 dB, with isolation > 14 dB without and > 25 dB with the structure. Figure 13b presents the 2D realized gain (θ = 90°, ϕ = 0°) and radiation efficiency.

**Fig. 14:**
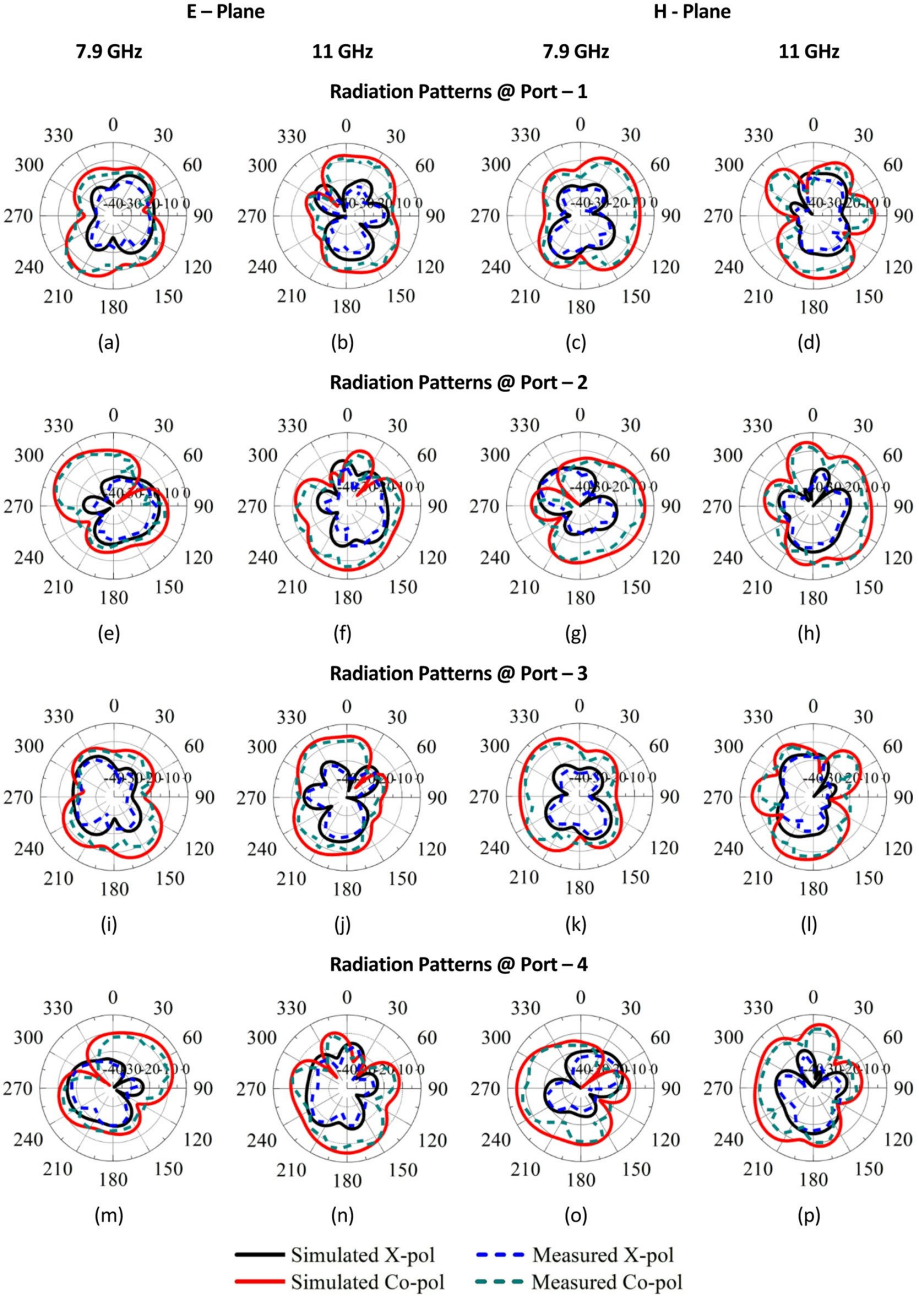
The radiation patterns of SRMPA for E-plane at 7.9 GHz is (**a**, **e**, **i**, **m**) and at 11 GHz (**b**, **f**, **j**, **n**); for H-plane at 7.9 GHz is (**c**, **g**, **k**, **o**) and at 11 GHz (**d**, **h**, **l**, **p**).

**Fig. 15:**
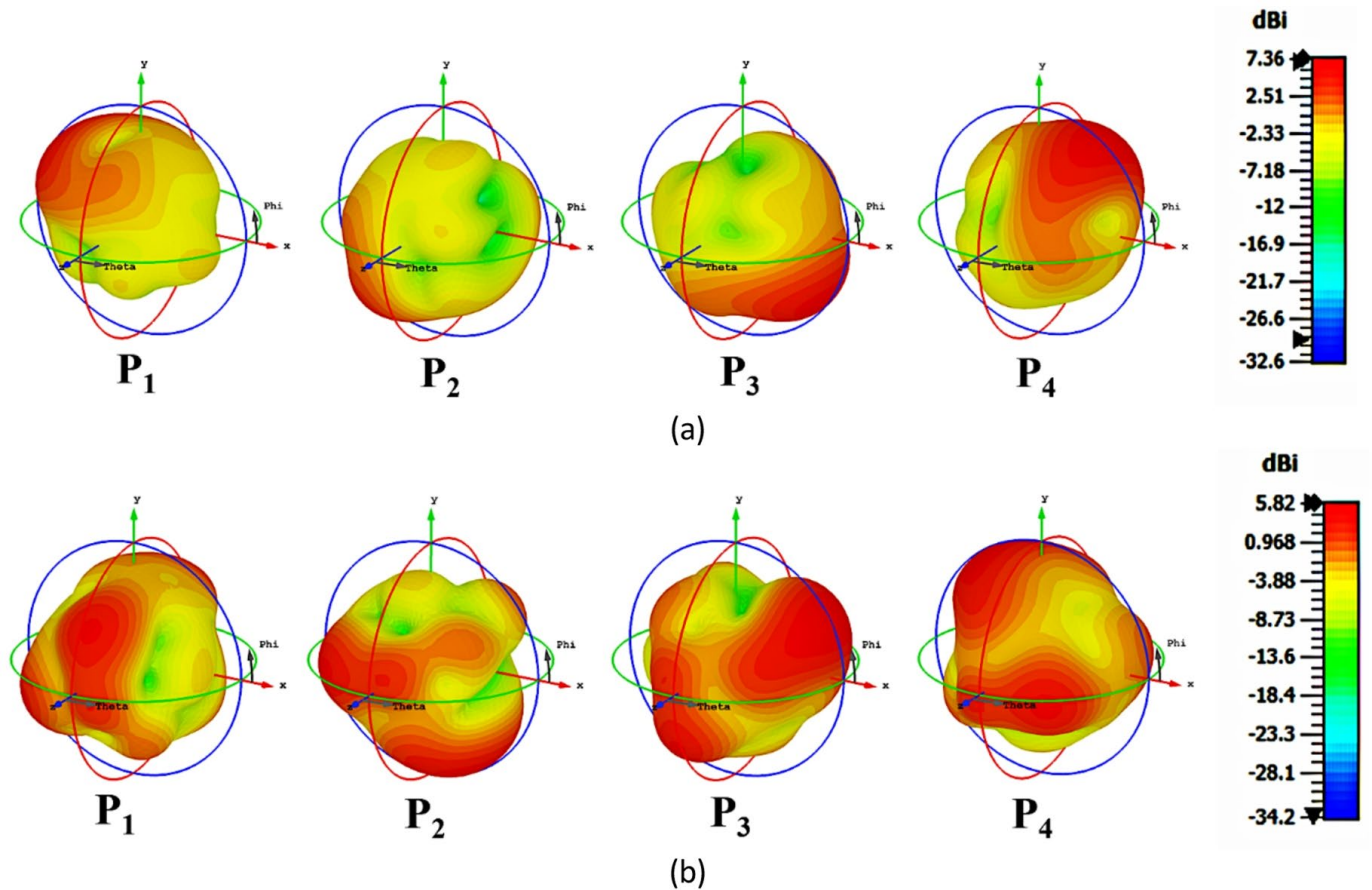
3D Gain characteristics of SRMP antenna at (**a**) 7.9 GHz, and (**b**) 11 GHz.

Figure 14a–p presents the simulated and practical E and H plane radiation patterns for co- and cross-polarization at P_1_-P_4_. 3D gain characteristics of SRMPA at operating frequencies for all ports (P1-P4) are shown in Fig. 15a, b. Figure 15a, b shows peak gains of 7.36dBi at 7.9 GHz and 5.82dBi at 11.0 GHz. The SRMPA results are validated in Table 4.

**Table 4:** Comparison of predicted and tested outcomes of SRMP antenna.

Validation	Resonant Frequency (GHz)	Gain (dBi)	ECC	DG (dB)	CCL (bits/s/Hz)	Isolation (dB)
Simulated	7.9 11.0	7.36 5.82	< 0.01	> 9.95	< 0.024	> 25
Measured	7.9 11.0	7.12 5.61	< 0.01	> 9.93	< 0.022	> 25

**Fig. 16:**
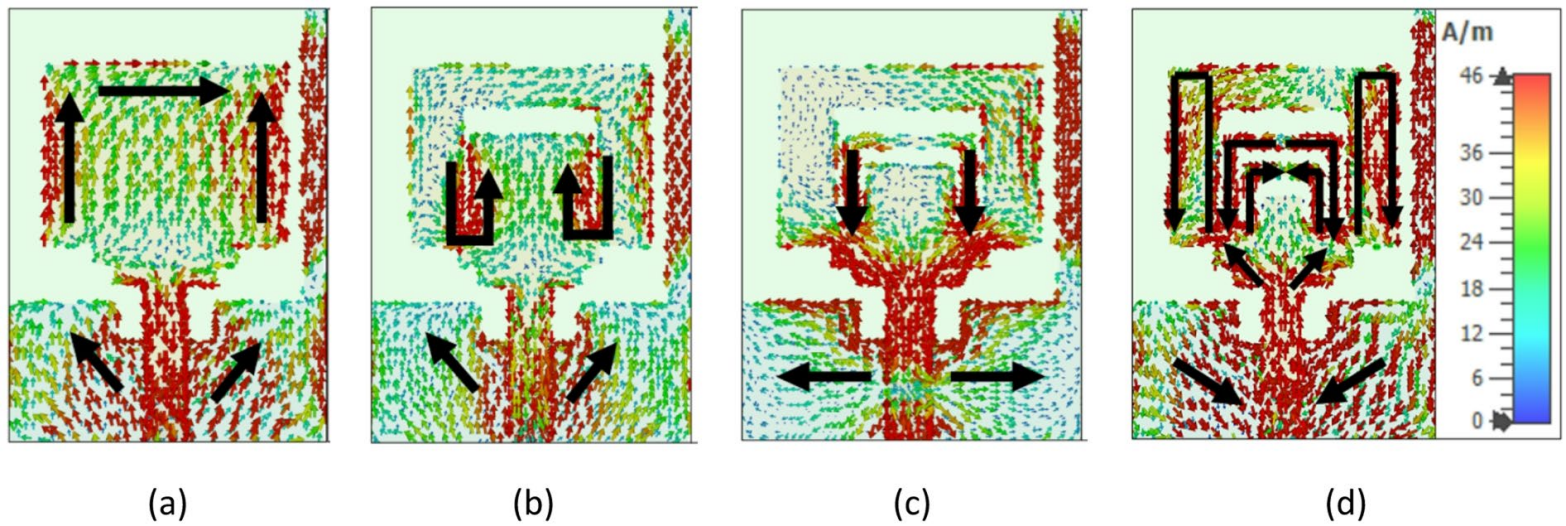
Surface current flow path of SRMP antenna with (**a**) Plain rectangular, (**b**) Single U-slot, (**c**) Double U-slots, and (**d**) Triple U-slots.

The surface current path of the SRMP antenna and role of each U-shaped slots on the patch are explained with help of unit element structure of the proposed SRMPA and it is shown below Fig. 16.

Step-by-step surface current patch flow is presented for four cases in Fig. 16a–d. Step-1 is plain rectangular patch is considered without U-shaped slots, Step-2 is etching of single U-shaped slot, Step-3 is etching of two U-shaped slots, and finally Step-4 we have consider etching of triple U-shaped slots (proposed antenna model of inverse U-shaped slots).

In Step-1 (without U-slot), the surface current primarily flows along the outer edges of the patch in a single loop, which supports only one dominant resonance, resulting in limited impedance matching, narrow bandwidth, and moderate performance. In Step-2 (with a single U-slot), the current begins to divide between the outer patch surface and the slot edges, thereby extending the effective current path length. This modification introduces an additional resonance, enabling dual-band operation while improving impedance matching and slightly enhancing bandwidth. In Step-3 (with two U-slots), the current distribution becomes more complex as it forms multiple nested loops along the slot boundaries, further increasing the electrical path length. This leads to stronger mode separation, better utilization of the radiating structure, improved isolation, and support for multiband operation. Finally, in Step-4 (with three U-slots), the current flow becomes highly confined and redirected along multiple parallel loops, producing the longest current paths and multiple well-defined resonant modes.

**Fig. 17:**
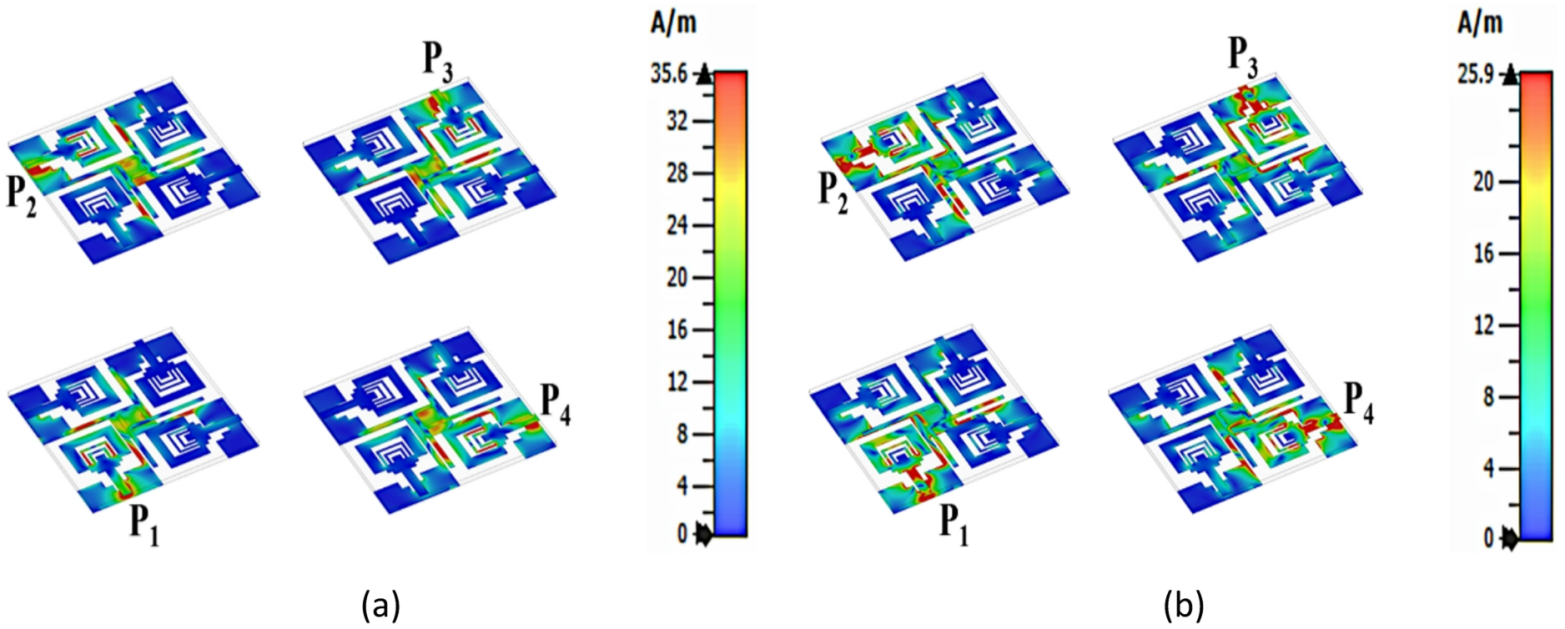
Surface current distribution of SRMP antenna when P_1_, P_2_, P_3_, and P_4_ are excited at (**a**) 7.9 GHz, (**b**) 11 GHz.

Figure 17a, b shows surface current distribution (SCD) with peak currents of 35.6 A/m at 7.9 GHz and 25.9 A/m at 11.0 GHz, concentrated at the edges of the U-shaped slot, slit on proposed SRMP antenna. The SCD presents with contour plots is presented in Fig. 17 at each port for dual bands.

Comparison of SRMPA with literature is listed in Table 5. SRMPA has high diversity characteristics and MIMO metrics for wireless communication. From comparison table SRMPA having very compact in size and area compared to^7^ and^22^. Also, the proposed antenna having high gain and wider impedance bandwidth characteristics compared to^7,9,16^, and^22^. The correlation between the elements is < 0.01 and diversity gain values of MIMO elements is greater than 9.95 these results are better compared to^16,22^, and^24^. The SRMP antenna having isolation between the port’s adjacent ports P_1_ and P_2_, and P_1_ and P_4_ are greater than 26 dB, opposite ports P_1_ and P_3_ is greater than 25 dB throughout the band which very high when compared to^2,7,16,24^. It indicates SRMPA is suitable for wireless application with better diversity characteristics.

**Table 5:** Comparison of SRMP antenna with recently published work.

Ref No	Antenna Size (λ × λ)	F_r_ (GHz)	Gain (dBi)	E-to-E Distance (λ_0_) (mm)	RBW (%)	ECC	Isolation (dB)	Applications
^2^	0.83 × 0.83	5.8	1.7	4	32	< 0.01	> 15	Wireless applications
^7^	2.52 × 2.52	7.20 14.5	3.60 6.60	4	34	< 0.01	> 20	Wireless applications
^9^	0.95 × 1.61	8.0 14.0	7.00	4	14	< 0.01	NA	Ku–band applications
^16^	3.44 × 1.59	13.2 16.6	4.00	6	21	< 0.02	> 24	Wireless applications
^22^	1.35 × 1.35	6.0 16.2	4.6 4.4	6	16	< 0.05	> 25	Wireless applications
^24^	1.13 × 0.94	7.8 14.2	NA	4	16	< 0.01	> 22	Wireless applications
Proposed Antenna	0.5 × 0.5	7.9 11.0	7.36 5.82	4	26	< 0.01	> 25	Wireless applications

NA, Not Available, Fr, resonating frequency, E-to-E distance, Edge-to-edge distance, RBW, relative bandwidth.

## Conclusion

A miniaturized staircase-shaped four-port MIMO patch antenna with a modified defected ground structure (MDGS) has been proposed for dual-band wireless communication applications. The orthogonal arrangement of the radiating elements, combined with a decoupling structure, ensures low mutual coupling and high isolation. The antenna’s performance has been validated through S-parameters, radiation patterns, gain measurements, and MIMO metrics. It achieves peak gains of 7.36 dBi and 5.82 dBi at 7.9 GHz and 11.0 GHz, respectively, with ECC < 0.01, DG > 9.95 dB, CCL < 0.02 bits/s/Hz, and MEG between − 6 dB and − 9 dB. Simulated and measured results show strong agreement, confirming the antenna’s effectiveness for compact wireless communication systems.

## Data Availability

The datasets used and/or analysed during the current study available from the corresponding author on reasonable request.
